# Supplementary material for: Phylogeny, Virulence, and Antimicrobial Resistance Gene Profiles of Enterococcus faecium Isolated from Australian Feedlot Cattle and Their Significance to Public and Environmental Health
Source: Antibiotics (Basel). 2023 Jun 28;12(7):1122. doi: 10.3390/antibiotics12071122 (PMC10376260; doi:10.3390/antibiotics12071122)
Supplement: Supplementary file 1 [file antibiotics-12-01122-s001.zip › antibiotics-2468424-supplementary.pdf]

Supplementary material

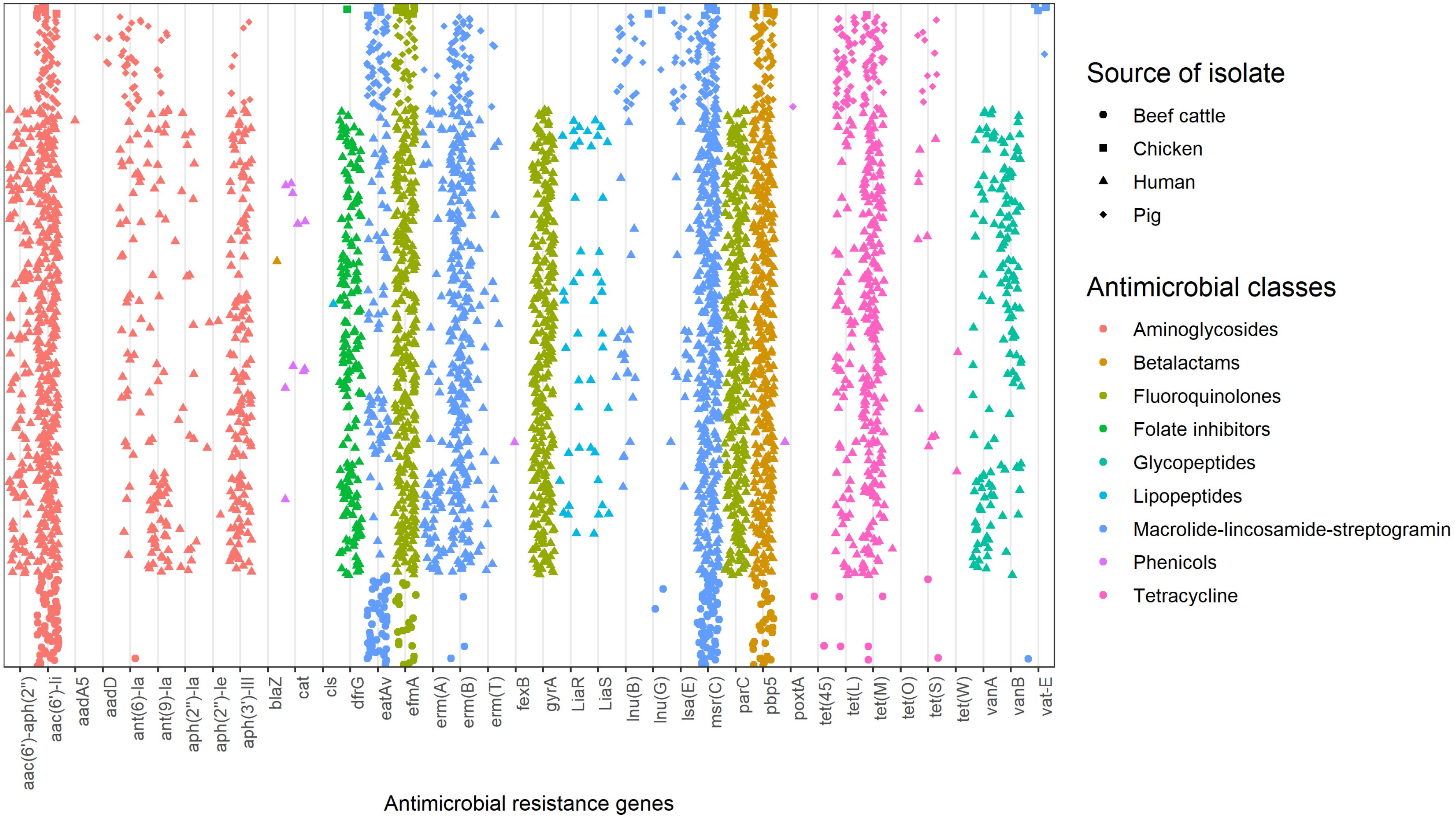

Figure S1. The frequency of antimicrobial resistance gene in *E. faecium* isolated from beef (n=59), pig (n=60), chicken (n=8) and human (n=302).

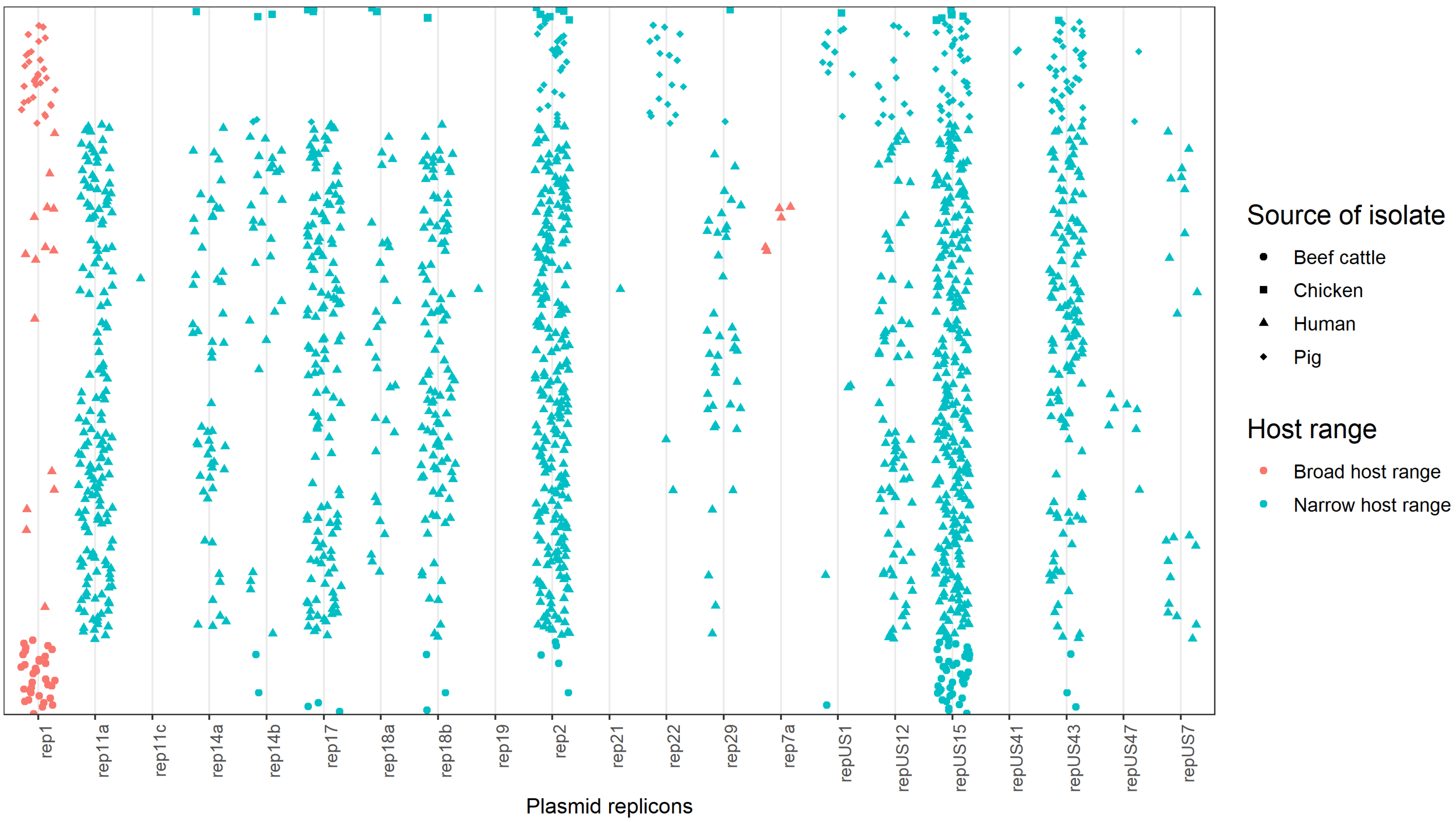

Figure S2. The frequency of plasmid replicons in *E. faecium* isolated from beef (n=59), pig (n=60), chicken (n=8) and human (n=302).

Table S1. Prevalence of antimicrobial resistance genes, plasmid replicons, and virulence genes in *E. faecium* (n=23) human Isolates mixed in animal isolate-dominated clades.

Human isolates (n=23)	MLST	Antimicrobial resistance genes	Plasmid replicons	Virulence genes
SRR10040962	94	*aac(6')-Ii, eatAv, msr(C)*	repUS15	*acm, efaAfm*
SRR10041064	1051	*aac(6')-Ii, eatAv, msr(C)*	rep1	*acm, efaAfm*
SRR10041102	994	*aac(6')-Ii, eatAv, msr(C)*	rep1, rep2, repUS15	*acm, efaAfm*
SRR10041153	1571	*aac(6')-Ii, eatAv, msr(C)*	rep1, repUS15	*acm, efaAfm*
SRR10041173	327	*aac(6')-Ii, eatAv, msr(C)*		*acm, efaAfm*
SRR10040882	855	*aac(6')-Ii, efmA, msr(C)*	rep1,repUS15	*acm, efaAfm*
SRR10040945	1565	*aac(6')-Ii, eatAv, msr(C), pbp5*	rep1, rep2, rep29, repUS15	*acm, efaAfm*
SRR10040968	800	*aac(6')-Ii, eatAv, msr(C), pbp5*	rep1, repUS15	*acm, efaAfm*
SRR10040975	361	*aac(6')-Ii, eatAv, efmA, msr(C)*	repUS15	*acm, efaAfm*
SRR10041049	717	*aac(6')-Ii, eatAv, msr(C), pbp5*	rep2	*acm, efaAfm*
SRR10041177	1556	*aac(6')-Ii, eatAv, msr(C), pbp5*	rep1, repUS15	*acm, efaAfm*
SRR10041104	22	*aac(6')-Ii, efmA, msr(C), pbp5*		*acm, efaAfm*
SRR10041106	1532	*aac(6')-Ii, efmA, msr(C), pbp5*	rep1, repUS15	*acm, efaAfm*
SRR10041117	22	*aac(6')-Ii, efmA, msr(C), pbp5*		*acm, efaAfm*
SRR10040901	32	*aac(6')-Ii, efmA, msr(C), pbp5*		*acm, efaAfm*
SRR10041023	1533	*aac(6')-Ii, efmA,msr(C), pbp5*	rep2, rep18a, repUS1	*acm, efaAfm*
SRR10041037	94	*aac(6')-Ii, eatAv, efmA, msr(C), pbp5*	repUS15	*acm, efaAfm*
SRR10041066	12	*aac(6')-Ii, eatAv, efmA, msr(C), pbp5*		*acm, efaAfm*
SRR10041086	1531	*aac(6')-Ii, eatAv, efmA, msr(C), pbp5*		*acm, efaAfm*
SRR10041091	94	*aac(6')-Ii, eatAv, efmA, msr(C), pbp5*	rep11c, repUS15	*acm, efaAfm*
SRR10040933	54	*aac(6')-Ii, efmA, eatAv, msr(C), pbp5*	rep1, repUS15	*efaAfm*
SRR10040988	1534	*aac(6')-Ii, ant(6)-Ia, eatAv, efmA ,erm(B), lnu(B), lsa(E),msr(C), pbp5, tet(L),tet(M)*	rep22, repUS12, repUS15, repUS43	*efaAfm*
SRR10040956	1535	*aac(6')-Ii, ant(6)-Ia, ant(9)-Ia, efmA, erm(A), erm(B), fexB, lnu(B), lsa(E),msr(C),pbp5, tet(L), tet(M)*	rep1, rep2, rep17, rep22, rep29, repUS12, repUS47	*acm, efaAfm*
